# Effects of Bridge-Shaped Microchannel Geometry on the Performance of a Micro Laminar Flow Fuel Cell

**DOI:** 10.3390/mi10120822

**Published:** 2019-11-27

**Authors:** Muhammad Tanveer, Kwang-Yong Kim

**Affiliations:** Department of Mechanical Engineering, Inha University, Incheon 22212, Korea; tanvir.ranjha@gmail.com

**Keywords:** laminar flow fuel cell, numerical model, bridge-shaped microchannel, power density, ohmic losses

## Abstract

A laminar flow micro fuel cell comprising of bridge-shaped microchannel is investigated to find out the effects of the cross-section shape of the microchannel on the performance. A parametric study is performed by varying the heights and widths of the channel and bridge shape. Nine different microchannel cross-section shapes are evaluated to find effective microchannel cross-sections by combining three bridge shapes with three channel shapes. A three-dimensional fully coupled numerical model is used to calculate the fuel cell’s performance. Navier-Stokes, convection and diffusion, and Butler-Volmer equations are implemented using the numerical model. A narrow channel with a wide bridge shape shows the best performance among the tested nine cross-sectional shapes, which is increased by about 78% compared to the square channel with the square bridge shape.

## 1. Introduction

Off-grid sensors and portable microelectronic devices are being used for security monitoring, environmental, and biological purposes, which all require miniaturized power sources. These power sources require long run times with high energy density. Micro fuel cells (MFCs) could be a good solution because of their unique characteristics, i.e., high energy efficiency, high energy density, eco-friendliness, and long run time [1]. MFCs are being used as hybrid system alongside batteries to achieve better lifetime [2].

Co-fabricating MFCs with electronic components facilitates miniaturization, reduces the number of interconnections, weight, and cost, and improves the signal integrity [3,4]. To utilize the fuels with high energy density, the integration and miniaturization of fuel cell systems are desirable. The miniaturization of the whole fuel cell systems is not merely de-escalating of a large-size fuel cell and its secondary components by using micro-fabrication techniques [2]. Micro-fabrication techniques are used to develop MFCs [5,6,7], which mainly utilizes proton exchange membrane (PEM). They are a potential power source for a variety of applications including portable electronics and MEMS devices [8,9,10,11,12].

However, major issues and key challenges have to be addressed. Multiple issues in current MFC designs are associated with the PEM [13,14,15,16,17]. It is challenging to achieve high conductivity for protons within the PEM for different operating conditions. The PEM requires water management, which adds further complications to the design of the MFC system. Another issue is fuel crossover through the PEM. Water uptake and dehydration also cause swelling and shrinkage and deform the PEM, resulting in packaging failure.

Ferrigno et al. [18] proposed a laminar flow fuel cell (LFFC) using parallel fuel and oxidant streams in the microchannel of an MFC. The electrochemical reactions, i.e., oxidation and reduction, take place at anode and cathode, respectively, by parallel fuel and oxidant streams down the microchannel. When the reactants are consumed closer to an electrode, a depletion region is formed near the electrode. In the LFFC, the PEM was removed, and the inter-diffusion zone between the two streams at the center of the channel acts as a membrane. The inter-diffusion zone at the interface between fuel and oxidant streams is referred as diffusive mixing zone or mixing region. The lower convective transportation of mass within laminar flows at smaller Reynolds numbers removes instabilities and allows streams to flow side-by-side with different concentrations in a microchannel. The diffusive mixing region along the interface between the two streams in the microchannel can be tailored to minimize crossover issues while allowing protons to move efficiently through the diffusion region [14,15,16]. Kjeang et al. [19] provided a detailed comparison of multiple LFFCs considering their performance, design, and fabrication.

The removal of the PEM in LFFCs significantly simplifies the operational design of MFCs, which assures significant miniaturization and inspires researchers and scholars to channel their efforts for the findings in research gap into on-chip integration [20,21]. The old model of an MFC stack comprising bipolar plates and electrode membrane assembly can be compounded in a single substratum, by ousting the PEM. LFFCs significantly reduce the Ohmic losses related to the PEM, and remove the issues related to sealing, water management, electro-osmotic drag, the hydration-dehydration cycle, and cathode flooding [22,23,24].

Numerous computational and experimental studies have been performed to find how the performance of LFFCs is affected by the flow rate, concentration, and channel geometry [25,26,27,28,29,30,31]. Tanveer and Kim [16] studied several geometric variations in a LFFC with Y-shaped channel. As the channel width, length, and height decreased, the power density increased. Many cross-sectional geometries and channel configurations were evaluated, and an LFFC model with the best performance was suggested. Tanveer and Kim [14] investigated multiple oxidant streams, the inlet placement, and multiple compartments of an LFFC. Utilizing multiple oxidant streams and locating the inlets for fuel and oxidant at the halfway point of the channel improved the LFFC performance significantly.

Shaegh et al. [32] and Krishnamurthy et al. [33] developed wide-range models for LFFCs and investigated numerous design related issues for LFFCs: i.e. fuel utilization, fuel crossover, and the impact of the electrode placement, structure, and geometric modifications on the fuel cell performance [2]. Montesinos et al. [15] proposed a μLFFC (micro laminar flow fuel cell) with a channel having a bridge-shape cross-sectional geometry, which minimized the reactant crossover. Montesinos et al. [34] investigated numerically a bridge structure for different concentrations and flow rates of the oxidant and fuel. They found that the use of higher flow rates and/or lower bridge aspect ratios reduced the width of the diffusive mixing zone, which reduced the diffusive crossover of the fuel. Tanveer and Kim [35] proposed multiple inlets (i.e., channels with four and eight inlets) in a μLFFC system, with a cross-sectional channel geometry of bridge shape. They found that increasing the number of inlets up to 8 caused an increase in the current density of about 15 in comparison with a 2-inlet μLFFC.

The present work investigated bridge-shaped microchannels for a μLFFC using three-dimensional numerical analysis to find out how the channel and bridge geometries affect the mixing and depletion zones and thus the μLFFC performance. The reaction kinetics, flow, and species concentration in the μLFFC were numerically modeled using the Butler-Volmer equation [36], three-dimensional Navier-Stokes equations, and the convection and diffusion equations. The model combines all the transport and electrochemical processes occurring within the μLFFC and was validated against the experimental results achieved by Montesinos et al. [15] Several new cross-sectional configurations of the μLFFC microchannel are proposed, and their impact upon the performance of fuel cell were studied.

## 2. LFFC Geometry 

**Figure 1:**
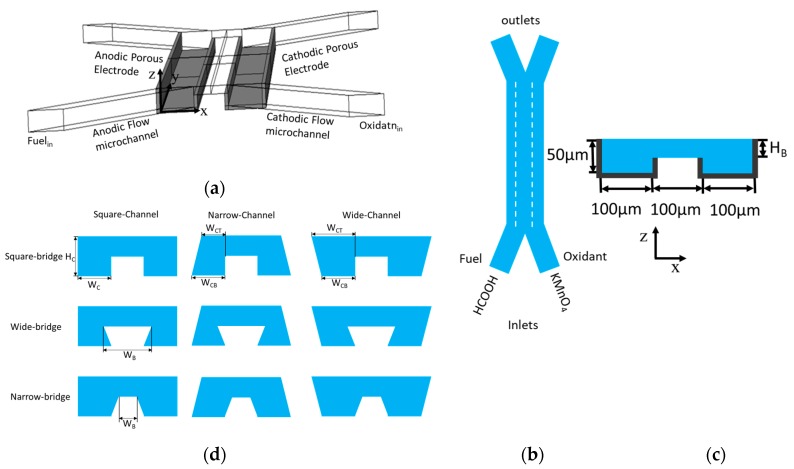
LFFC microchannel configurations: (**a**) three-dimensional view and electrode locations (dark regions); (**b**) two-dimensional view of microchannel; (**c**) cross-sectional view of bridge-shaped channel (the bold outlines indicate electrodes); (**d**) nine cross-sectional geometries of microchannel.

Figure 1 shows the tested microchannel configurations. The cross-sectional geometry of the μLFFC microchannel of bridge-shape was proposed by Montesinos et al [15]. This configuration paved a way toward reducing the fuel-oxidant interfacial contact. It also provides an option for efficient proton transportation by isolating the depletion region from the diffusive mixing region. The isolation or minimal interaction of these two regions allows the efficient reactions at the anode and cathode. The bridge-shaped cross-section provides the electrodes with multiple sides, which expose the fuel and oxidant to a larger reactive area. 

There are two channels: anodic and cathodic flow channels. A bridge connects these two channels, as shown in Figure 1. The bridge width (*W*_B_), channel width (*W*_C_), bridge height (*H*_B_), and channel height (*H*_C_) shown in Figure 1c are the geometric parameters to be changed. The widths of the channel base and top (*W*_CB_ and *W*_CT_, respectively) are different except in the case of the square-bridge square-channel shape, and they were varied to define eight different configurations proposed in this work, as shown in Figure 1d. The microchannel length is 10 mm. The narrow-channel and wide-channel configurations were obtained by uniformly shortening and extending the width of the top of the channel (*W*_CT_) by 25 µm from both sides of the square channel, respectively, as shown in Figure 1d. Similarly, narrow-bridge and wide-bridge configurations were obtained by uniformly shortening and extending the bridge-width (*W*_B_) by 25 µm from both sides of the square-bridge, respectively. The validation of numerical model was performed for a reference μLFFC with a square-bridge square-channel shape with H_B_ = 10 µm, H_C_ = 50 µm, W_C_ = 100 µm (= W_CB_ = W_CT_), W_B_ = 100 µm, and a 60 μL/min flow rate.

## 3. Numerical Analysis

A low Reynolds number occurs due to the micro scale dimension of the μLFFCs and makes the flow laminar. The internal heating due to viscous dissipation is neglected. Therefore, the system is considered incompressible and isothermal. For the numerical analysis, several assumptions were made: Newtonian flow, steady flow, no body force, constant fluid densities, and constant temperature. The depletion region was assumed to have minimal or no impact on the mixing zone and local concentration gradient, even at the outlet where the widest thickness of the mixing zone occurs. A CFD code based on the finite element method, COMSOL Multiphysics 4.3b [37] was used to analyze the performance of the μLFFC.

The flow analysis was carried out using the continuity and Navier-Stokes equations:

(1) ∇·u=0

(2) $$\rho \left(u\cdot \nabla u\right)=-\nabla p+\mu \nabla^{2}u$$

Here, p, u, and ρ are the pressure, vector velocity, and fluid density, respectively. The transportation of species is governed by convection and diffusion, which is represented by the following convection-diffusion equation:

(3) ∇·(−D∇c+cu)=0

where D and c are the diffusivity and the concentration, respectively.

To calculate the concentration and velocity fields, the equations are solved simultaneously with the appropriate boundary conditions (BCs). Zero-pressure conditions are applied at the outlet. No-slip BC (u = 0) were assigned at the channel walls. Convective mass flux was applied at the outlet, and at inlets a constant concentration was specified. The mass transportation is neglected through the channel walls, hence the equation becomes:

(4) n·(−D∇c+cu)=0

The fuel and oxidant are formic acid and potassium permanganate, respectively. The anodic electrochemical reaction is as follows:

(5) $$HCOOH\to CO_{2}+2H^{+}+2e^{-}$$

The primary permanganate reduction at the cathode in acidic medium is as follows:

(6) $$MnO_{4}^{-}+8H^{+}+5e^{-}\to Mn^{2+}+4H_{2}O$$

The permanganate ion (Mn^2+^) is further oxidized into insoluble MnO2 by the following reaction:

(7) $$2MnO_{4}^{-}+3Mn^{2+}+2H_{2}O\to 5MnO_{2}+4H^{+}$$

The Butler-Volmer equation and Faraday’s law are used to model the electrochemical reactions. Oxidant and fuel (KMnO_4_ and HCOOH, respectively) are used after mixing with acidic electrolyte, and the hydrogen ions’ concentration is supposedly uniformly distributed all over the microchannel. The migration defines the transportation of hydrogen ions from the anodic flow channel to the cathodic flow channel. The current collector and catalytic layers are modeled within the anode and cathode electrodes as a solid-phase porous sub-domain. The effective conductivity value is calculated using Archie’s law [38,39] in the porous electrodes.

To accommodate the electrode reactions, the mass transport equations are updated. For the anodic flow channel and cathodic flow channel, the transportation of species is defined as follows:

(8) ∇·(−D∇c+cu)=R

where the rate of reaction (R) is considered zero, and the reactions at reactive sides can be modeled using fluxes. These fluxes can be described as:

(9) $$-n\cdot N=N_{0}$$

(10) N=−D∇c+cu

where n and N are the normal vector and flux vector, respectively. *N*_0_ is the flux of the oxidant or fuel consumption at the electrode walls. 

The oxidant and fuel consumed by the reaction over anode or cathode, are computed by Faraday’s law:

(11) $$N_{0}=\frac{J_{n}}{e_{i}F}$$

wherein $e_{i}$, *J_n_*, and F are the number of moles involved, the local current generated at the electrodes in normal direction by electrochemical reactions, and the Faraday constant, respectively. *J_n_* is zero except for reactive walls.

The charge conservation used to compute the current transportation is either ionic or electronic:

(12) $$\nabla \cdot \left(-\sigma \nabla \varphi \right)=A_{s}S_{\varphi }$$

where *S_φ_*, *φ*, *σ*, and *A_s_* are the source term for the electric current, local potential, conductivity of the layer, and the electrochemical surface area, respectively. When the volumetric terms are assumed zero, the equation is reduced to the following:

(13) ∇·(−σ∇φ)=0

The current originating from solid-phase porous electrodes can be computed by adapting the charge conservation equation as follows:

(14) $$\sigma_{eff}=\sigma_{0}{\left(1-\varepsilon \right)}^{m}$$

where ε is the porosity, and σ_0_ is the non-porous material’s electrical conductivity. The value of m varies within 1.8 to 2.5 [1,2]. 

At the anode, the solid-phase potential (*φ*_s_) is set as zero and it is the operating cell voltage (Vcell) for the cathode. The boundary conditions at porous electrode used to determine the current generated are as follows:

(15) $$-n\cdot J=J_{n}$$

where *J_n_* and J being the normal local current and vector current. *J_n_* is calculated using the Butler-Volmer equations:

(16) $$J_{n_{a}}=i_{0}c/c_{ref}\left\{exp\left(-F\eta_{a}\frac{\alpha_{a}}{RT}\right)-exp\left(-F\eta_{c}\frac{\alpha_{c}}{RT}\right)\right\};c=c_{HCOOH\;}anode$$

(17) $$J_{n_{c}}=i_{0}c/c_{ref}\left\{exp\left(-F\eta_{c}\frac{\alpha_{a}}{RT}\right)-exp\left(-F\eta_{c}\frac{\alpha_{c}}{RT}\right)\right\};c=c_{KMnO_{4}}\;cathode$$

where $i_{0}$, η, T, R and α are the exchange current density, overpotential, temperature, ideal gas constant, and charge transfer coefficient, respectively. The subscripts c and a denote the cathode and anode, respectively. *c_ref_* and *c* are the reference concentration and local reactant’s concentration, respectively. The overpotentials at anode and cathode are defined as:

(18) $$\eta_{a}=\varphi_{s}-\varphi_{e}-\varphi_{rev_{a}}\;;\;anode$$

(19) $$\eta_{c}=\varphi_{s}-\varphi_{e}-\varphi_{rev_{c}}\;;\;cathode$$

where *φ_e_* is the liquid-phase potential, and *φ_rev_* is the reversible potential.

## 4. Results and Discussion

**Figure 2:**
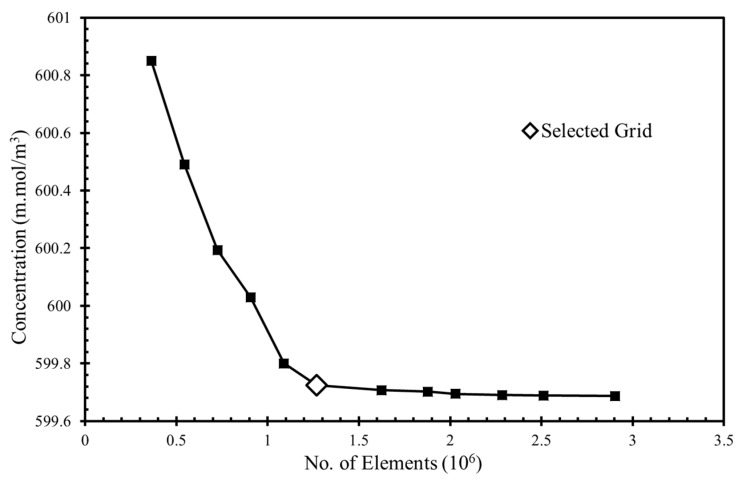
Test of grid independency.

To obtain grid-independent solutions, a grid refinement test was performed. Several mesh element numbers in the range of 3.6 × 105 to 2.9 × 106 were tested for the fuel’s concentration at the the microchannel outlet (Figure 2). The grid was created by using the COMSOL default physics controlled mesh option, which created finer and coarser elements at the boundary layers and the bulk domain, respectively. The concentrations were found using the “dilute species’ transportation” module in COMSOL Multiphysics. [37] There was a minimal 0.0028% relative change in the concentration between the grids with 1.27 × 10^6^ and 1.62 × 10^6^ element, so the grid with 1.27 × 10^6^ elements was chosen for additional calculations.

**Figure 3:**
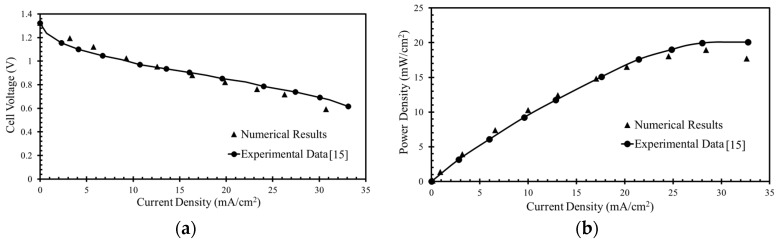
Comparison of the numerical results: (**a**) cell voltage vs. current density; (**b**) power density vs. current density with experimental data of Montesinos et al. [15] for validation (Tanveer and Kim [35]).

The numerical model was validated previously [35] by comparison with experimental results achieved by Montesinos et al. [15], as presented in Figure 3. Variations in the cell voltage against current density and power density with the current density were found for a µLFFC with the microchannel having bridge-shaped cross-section with a 100-µm bridge height (*H*_B_), 50-µm channel height (HC), and 100-µm bridge width (*W*_B_) and channel width (*W*_C_). To carry out the calculations, concentrations of 144 mM KMnO_4_ (oxidant) and 1 M HCOOH (fuel) were taken. 

The numerical results exhibit good agreements with the experimental data, as shown in Figure 3. The numerical results were found to deviate a little from the experimental results at higher current densities for both the cell voltage and peak power density. The reason might be insoluble MnO_2_ at lower cell voltages. Therefore, the mass transportation losses reduce the overall performance at lower cell voltages at the cathode. The model conforms well with the experimental data at cell voltages in the range of 0.6–0.8 V, as shown in Figure 3a, and fuel cells are operated practically at these voltages. Therefore, the results are reported for this range of cell voltages (voltages larger than 0.5 V). The cell voltage (*V*_cell_) is the solid-phase potential assigned to the cathode electrode for each point on the polarization curve (the voltage plotted against the current density), and the anode is arbitrarily grounded. Thus, the μLFFC performance is mainly limited by the cathode.

The potential equation’s BCs are given at the anode or cathode sub-domain. To calculate the current produced by the μLFFC, the local current being produced normal to the cathode is integrated over the whole cathodic walls. Then cell voltage is multiplied by the current density result in power density.

**Figure 4:**
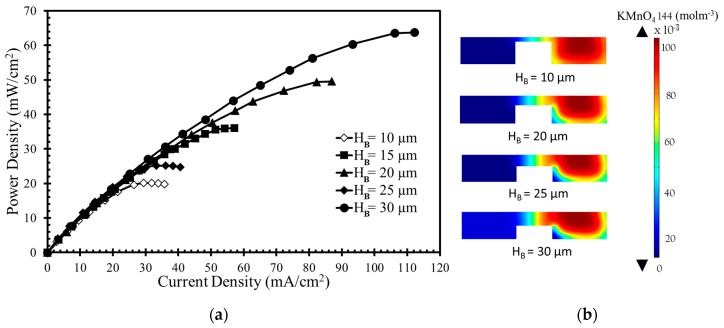
Effects of bridge height on power density curve: (**a**) performance curve; (**b**) Concentration contours of oxidant (KMnO_4_) at the x-z plane 10 mm downstream of the active channel for bridge height.

**Figure 5:**
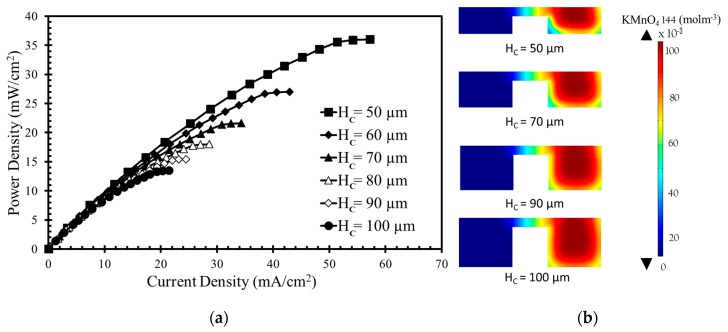
Effects of channel height on power density curve: (**a**) performance curve; (**b**) Concentration contours of oxidant (KMnO_4_) at the x-z plane 10 mm downstream of the active channel for channel height.

**Figure 6:**
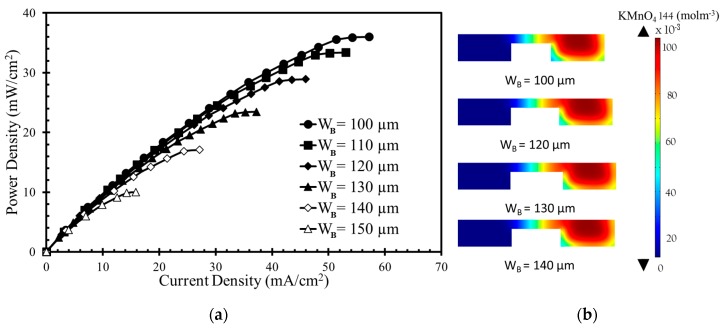
Effects of bridge width on power density curve: (**a**) performance curve; (**b**) Concentration contours of oxidant (KMnO_4_) at the x-z plane 10 mm downstream of the active channel for bridge width.

**Figure 7:**
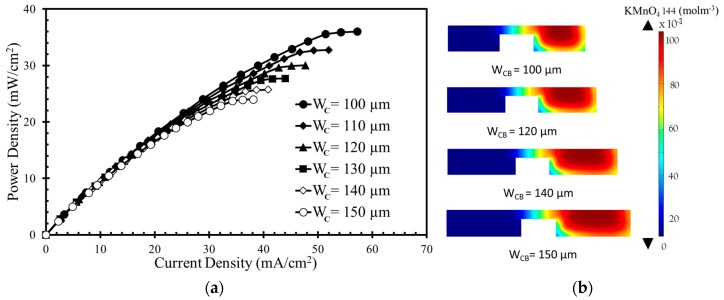
Effects of channel width on power density curve: (**a**) performance curve; (**b**) Concentration contours of oxidant (KMnO_4_) at the x-z plane 10 mm downstream of the active channel for channel width.

The numerical model was further used to investigate the effects of the width and height of the bridge and channel on the performance of the μLFFC with a square bridge and square channel. Figure 4, Figure 5, Figure 6 and Figure 7 shows the resulting power-density curves and corresponding concentration contour plots. The following parametric values were used in these tests: *H*_B_ = 20 µm, *H*_C_ = 50 µm, *W*_C_ = 100 µm, *W*_B_ = 100 µm.

Figure 4a shows the results for various bridge heights. Increasing the bridge height results in higher power density and current density due to the increased bridge height leading to the passage of more ions through the diffusive mixing zone, which reduces Ohmic losses. The diffusive mixing region becomes thinner with increased bridge height, and the depletion region becomes thicker with increased oxidant utilization at the cathode surface.

In Figure 4b the concentration contours of the oxidant (KMnO_4_) are plotted on the x-z plane located 10 mm downstream of the active channel. The contours are plotted for four different bridge heights (10, 20, 25, and 30 µm) in Figure 4b, which shows that higher bridge height leads to a larger depletion zone. Thinner diffusive mixing and depletion zones result in a higher concentration gradient in the bridge structure with less height, as shown in Figure 4b. The thinner depletion zone ensures a higher reaction rate to drive fresh reactant toward electrode. The efficient consumption of reactants widens the depletion zone and increases mass-transport losses. A thinner mixing zone ensures more efficient proton transport, which leads to higher current generation. Among the various bridge heights, *H*_B_ = 30 µm gives the highest power density approximately equal to 63 mW/cm^2^, as presented in Figure 4a. When *H*_B_ is varied from 10 µm to 30 µm, the increase in power density occur from 20 mW/cm^2^ to 63 mW/cm^2^ (by around 215%). Increasing the bridge height reduces Ohmic losses but is not effective in overcoming mass-transport losses.

The channel height was varied while keeping the other geometric parameters constant as reference values. The results in Figure 5a show that an increased channel height results in lower power density and current density. This is due to the increased channel height with enlarged surface area of the electrode reducing the mass-transport losses and increasing Ohmic losses. Higher electrode area plays an important role in reducing the power density. The diffusive mixing region remains almost the same, but the depletion region becomes thinner with increasing channel height, as shown in Figure 5b, where the concentration contours of the oxidant are shown for four different channel heights (50, 70, 90, and 100 µm). Among the channel heights, *H*_C_ = 50 µm with *H*_B_ = 20 µm yields the highest power density approximately equal to 36 mW/cm^2^, as given in Figure 5a. When H_C_ is varied from 50 µm to 100 µm, the power density decreases from 36.0 mW/cm^2^ to 13.5 mW/cm^2^ (approximately equal to 63%).

Figure 6a shows the effect of the bridge width on the power density curve. As the bridge width increases, the power density and current density decrease. The concentration contours of the oxidant are shown in Figure 6b for four different bridge widths (100, 120, 130, and 140 µm). Increasing the bridge width does not remarkably affect the diffusive mixing and depletion zones. As the bridge width increases, the thickness of both the zones remains almost un-changed, but the Ohmic losses increase because of the increased anode-to-cathode distance. The opposition to the movement of H+ (*Rf* = *d*/*σA*) increases as anode to cathode distance (d) increases. Here, A and σ are the electrodes’ area and the electrical conductivity, respectively. Among the bridge widths, *W*_B_ = 100 µm generates the highest power density approximately equal to 36 mW/cm^2^, as given in Figure 6a. When H_C_ is varied from 50 µm to 100 µm, the decrease in power density occurs from 36 mW/cm^2^ to 10 mW/cm^2^ (approximately equal to 72%).

Figure 7a shows that the power and current densities decreases as the width of the anodic or cathodic flow microchannel (*W*_C_) increases for the square-channel square-bridge shape (Figure 1c). The concentration contours of the oxidant are shown in Figure 7b for four different channel widths (100, 120, 140, and 150 µm). Increasing the channel width does not significantly affect the diffusive mixing zone. The thickness of the depletion zone decreases as the channel width increases. The power density curve shows that there is less of a decrease in the power density when the channel width increases compared to the other parameters. Among the channel widths, *W*_C_ = 100 µm generated the highest power density approximately equal to 36 mW/cm^2^, as given in Figure 7a. When W_C_ changes from 100 µm to 150 µm, the power density decreases from 36 mW/cm^2^ to 24 mW/cm^2^ (approximately equal to 33%).

The results of the parametric study shown in Figure 4, Figure 5, Figure 6 and Figure 7, give insight into how to minimize the losses (Ohmic and mass-transport losses). Modification of the bridge shape (i.e., the diffusive mixing zone) affects the mixing zone and thus the Ohmic losses. Modification of the microchannel cross-section, which supports the electrodes (the depletion region formed over electrode surface), affects the mass-transport losses.

**Figure 8:**
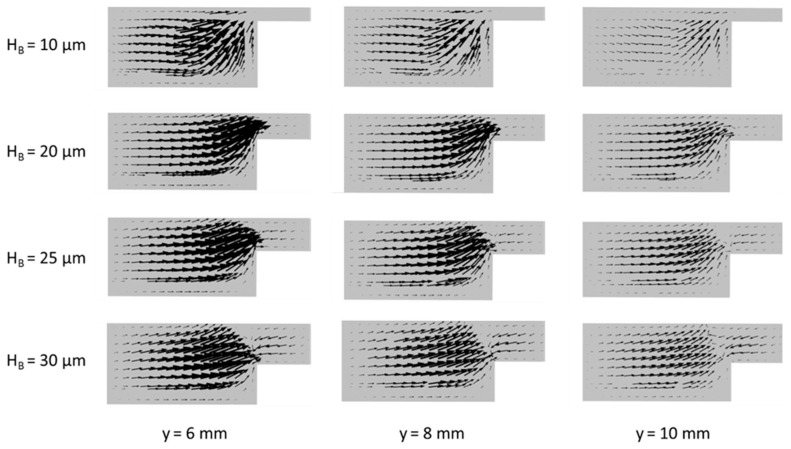
Velocity vector plot on x-z planes at 6, 8, and 10 mm downstream of the active channel inlet for various bridge heights.

Fuel crossover occurs because of the leakage of fuel toward the cathodic flow microchannel. Figure 8 shows velocity vector plots of the mixed fluid particles on the x-z planes at 6, 8, and10 mm downstream from the active channel inlet for various bridge heights. The momentum equations (Equation (2)) are solved only for the mixed fluid in this work. It is found in Figure 8 that the transverse flow in x-z plane becomes inactive as the flow proceeds downstream regardless of the bridge height. A smaller bridge height causes a thicker mixing region, as shown in Figure 5a, which prevents the leakage of fuel and oxidant from either side of the channel. When the bridge height is 10 µm, the magnitude of the velocity vectors through the bridge is negligible, as shown in Figure 8. But, as the bridge height increases, the velocities in the bridge increase. In all the cases, reverse flows are found in the bridge. This means that the mixing region at the center of the bridge acts like a barrier for the movement of fluid particles. However, the higher fluid velocity in the bridge indicates a higher possibility for the crossover of fuel or oxidant through the mixing layer. Nevertheless, the power density increases as the bridge height increases as shown in Figure 4a, because the positive effects of increasing ion transfer and reducing Ohmic losses on the power density exceed the negative effect of increasing crossover.

**Figure 9:**
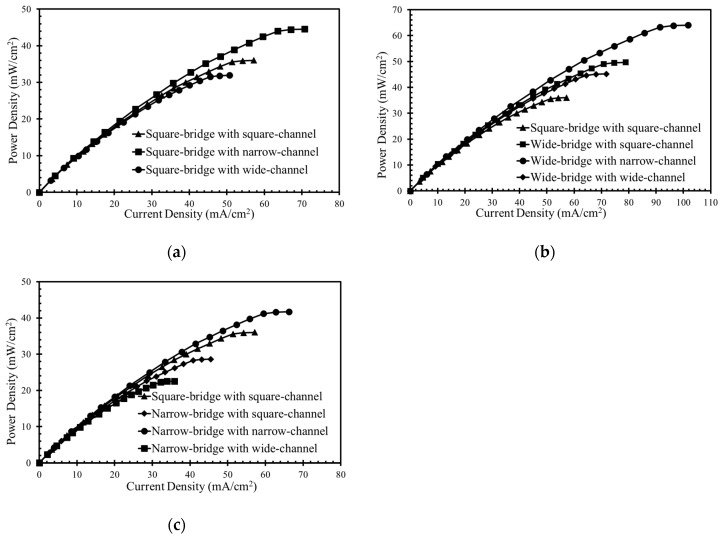
Effects of channel cross-sectional shapes on power density curve: (**a**) square bridge, (**b**) wide bridge, and (**c**) narrow bridge (shown in Figure 1d).

**Figure 10:**
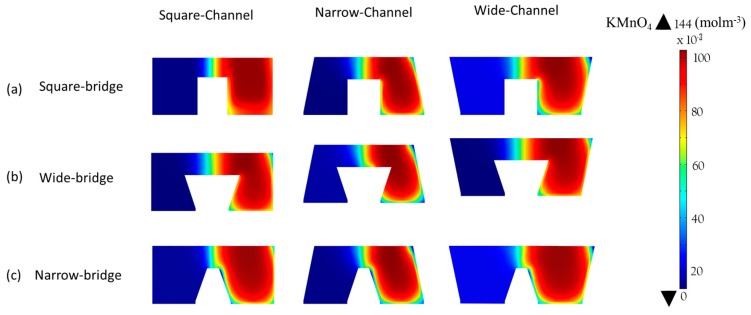
Concentration contours of oxidant (KMnO_4_) at the x-z plane 5 mm downstream of the main channel: (**a**) square bridge, (**b**) wide bridge, and (**c**) narrow bridge.

The performance of the microchannel cross-sectional shapes shown in Figure 1d was compared, as shown in Figure 9, where the power-density curves are presented. In Figure 10, the concentration contours of the oxidant are plotted, on the x-z plane at 5 mm downstream of the active channel for the nine different cross-section shapes.

Figure 9a shows the curves for the square bridge shapes, where the electrodes are placed on the channel walls, as shown in Figure 1c. The wide channel with a square bridge shape (Figure 1d) shows a decrease in maximum power density by about 13% compared to the square channel with a square bridge shape (Figure 9a). Increasing the width of the channel and electrode surface area reduces the maximum power density from 36 mW/cm^2^ to almost 32 mW/cm^2^. The narrow channel with a square bridge shape exhibits approximately 24% higher power density than the square channel with a square bridge shape. Reducing the width of the channel enhances the power density from 36.0 to 44.5 mW/cm^2^. Figure 10a shows the concentration contours for the square bridge shapes. Wide and narrow channels with a square bridge do not effectively reduce the depletion and mixing regions to improve the performance, as shown in Figure 10a. Therefore, the improvement in the performance is not pronounced for these shapes.

Figure 9b shows the effect of a wide bridge shape on the power-density curve. The concentration contours of the oxidant for the wide bridge shapes are shown in Figure 10b. The square channel with a wide bridge shape (Figure 1d) shows an increase in power density by about 38% compared to the square channel with a square bridge shape, as shown in Figure 9b. The wide bridge shape prevents the depletion region from merging with the diffusive mixing zone and hence reduces the ohmic and mass-transport losses effectively, as shown in Figure 10b. The wide channel with the wide bridge shape shows an increase in the maximum power density by about 25.5% compared to the square channel with a wide bridge shape (Figure 9b). Increasing the channel width increases the ohmic losses, resulting in a reduction of the power density. However, this reduction can be compensated for by increasing the bridge width. Extending the width of the channel enhances the maximum power density from 36.0 mW/cm^2^ to 45.2 mW/cm^2^, as shown in Figure 9b. 

The narrow channel with wide bridge shape is the most effective configuration for controlling the mass-transport and ohmic losses. Making the microchannel narrower and extending both sides of the bridge, reduces the ohmic losses in multiple ways. Making the microchannel narrower brings the electrodes closer and makes ion transfer more efficient. Extending the bridge on both sides ensures a sufficient gap between the depletion and diffusive mixing region, as shown in Figure 10b. A lower width of the channel supported by a wide bridge shape exhibits an enhancement in power density from 36 mW/cm^2^ to 64 mW/cm^2^ (approximately equal to 78%) in comparison with the square channel with a square bridge shape, as shown in Figure 9b.

Figure 9c shows the power-density curves for the narrow bridge shapes shown in Figure 1d. The concentration contours for the microchannels with a narrow bridge shape are shown in Figure 10c. The narrow channel with a narrow bridge shape exhibits an improvement of almost 16% in comparison with the square channel with the square bridge shape, as shown in Figure 9c. The narrow bridge shapes with square and wide channels do not show effective performance. In the channels with a narrow bridge shape, the depletion zone becomes close to the diffusion zone, as shown in Figure 10c. The contact of the mixing zone with the depletion zone causes crossover problems and increases ohmic losses and should thus be avoided in the μLFFC design. A narrow bridge shape combined with square and wide channels shows about 21% and 39% decreases in the power density, respectively, compared with the square channel combined with a square bridge.

Table 1 compares the performance of the LFFC with narrow channel with wide bridge which shows the best performance among the tested cross-sectional channel geometries, with those of previously developed LFFCs [15,22,26,27,29]. The cross-sectional shape of the microchannel proposed in this work shows a current density of 102 mA/cm^2^ which is far better than the others.

## 5. Conclusions

This study evaluated the effects of the width and height of the channel and bridge of a bridge-shaped microchannel cross-section on the performance of a μLFFC using a three-dimensional fully coupled numerical model. The main objective was to find a configuration that reduces the mixing and depletion zones to increase the performance without fuel crossover. Nine different microchannel cross-section shapes were evaluated. These shapes were obtained by combining three bridge shapes (square, wide, and narrow) with three channel shapes (square, wide, and narrow). The results show that the maximum power density decreased as the channel height, channel width, and bridge width increased, while it increased with the bridge height.

Among the proposed microchannel cross-section shapes, the narrow channel with the wide bridge shape was proven to be an effective choice for μLFFCs. Using this configuration, the maximum power density of the μLFFC increased from 36 mW/cm^2^ to 64 mW/cm^2^ (by about 78%) compared to the square channel with the square bridge shape. Although the actual performance depends on the particular fuel cell configuration, the predicted trends of the microchannel cross-sections are expected to be applicable to various configurations of μLFFCs.

The bridge-shaped microchannel is known to reduce the fuel to oxidatn interfacial, and thus isolates the depletion zone from the diffusive mixing zone. The extended bridge with a shortened microchannel further reduced the reactant crossover issue by separating the mixing and depletion zones. This helped to increase the reactant consumption by decreasing the ohmic and mass-transport losses and thus improving the overall fuel cell performance.

## Figures and Tables

**Table 1:** Comparison among LFFCs with different microchannel cross-sections.

Reference	Microchannel Cross-Section	Fuel/Oxidant	Current Density	Power Density
Choban et al. [22]	Square shaped	HCOOH/KMnO_4_	4 mA/cm^2^	2.4 mW/cm^2^
		HCOOH/O_2_	0.4 mA/cm^2^	0.17 mW/cm^2^
Bazylak et al. [27]	Rectangular shaped	HCOOH/O_2_	1.1 mA/cm^2^	
Khabazi et al. [29]	Extended square shaped	HCOOH/O_2_	0.32 mA/cm^2^	
	High aspect ratio		0.42 mA/cm^2^	
	Low aspect ratio		0.45 mA/cm^2^	
Park et al. [26]	H-shaped	HCOOH/KMnO_4_	34 mA/cm^2^	13 mW/cm^2^
Montesinos et al. [15]	Bridge-shaped	HCOOH/KMnO_4_	28 mA/cm^2^	26 mW/cm^2^
Present work	Narrow-channel with wide-bridge	HCOOH/KMnO_4_	102 mA/cm^2^	64 mW/cm^2^

## Nomenclature

$\eta_{a}$	Anodic overpotential (V)
$\eta_{c}$	Cathodic overpotential (V)
*ϕ*	Local-potential (V)
*V_cell_*	Operating cell voltage (V)
$\varphi_{s}$	Local-potential of the solid phase (V)
$\varphi_{e}$	Local-potential of the liquid phase (V)
$\varphi_{a}$	Anodic reversible-potential (V)
$\varphi_{c}$	Cathodic reversible-potential (V)
$i_{0}$	Exchange-current density of anode (A/m^2^)
$\alpha_{c}$	Charge transfer coefficient of cathode
$\alpha_{a}$	Charge transfer coefficient of anode
*U_y_*	Average flow velocity (m/s)
*c*	Species’ concentration (mol)
*σ*	Conductivity (S/m)
$I_{co_{f}}$	Crossover-current by fuel crossover (A)
$I_{co_{o}}$	Crossover-current by oxidant crossover (A)
J	Vector current
$J_{n}$	Normal current
$J_{n_{a}}$	Anodic normal current
$J_{n_{c}}$	Cathodic normal current
$I_{a}$	Anodic current (A)
$I_{c}$	Cathodic current (A)
*D*	Species’ diffusivity (m^2^/s)
*D_HCOOH_*	Fuel (HCOOH) diffusivity (m^2^/s)
*ρ*	Fluids’ density (kg/m^3^)
*μ*	Fluid viscosity (Pas)
*σ_eff_*	Effective conductivity (S/m)
*σ_0_*	Non-porous material’s Electrical conductivity (S/m)
$\sigma_{a}$	Electrical conductivity in anodic microchannel for liquid electrolyte (S/m)
$\sigma_{c}$	Electrical conductivity in cathodic microchannel for liquid electrolyte (S/m)
*A_s_*	Electrochemical surface area (m^2^)
$S_{\varphi }$	Source term for electrical current
*F*	Faraday constant (C/mol)
*R*	Ideal gas constant (J/mol·K)
N	Flux vector
n	Normal vector
$\dot{V}$	Flow rate (m^3^/s)
*L*_0_	Length of microchannel (m)
*H*	Channel Height (m)
Δx	Width of mixing zone (m)
u	Velocity vector (m/s^−1^)
*P*	Pressure (Pa)
*p_in_*	Inlet pressure (Pa)
*c_in_*	Inlet concentration (mol)
$M_{o}$	Molar flux for oxidant crossover
$M_{f}$	Molar flux for fuel crossover
*e_i_*	Number of moles
*ε*	Porosity
*c_ref_*	Reference concentration (mol)
*T*	Temperature (K)

## References

[1] Moghaddam S., Eakkachai S.P.W., Masel R.I., Shannon M. (2010). An enhanced microfluidic control system for improving power density of a hydride-based micro fuel cell. J. Power Sources.

[2] Shaegh S.A.M., Nguyen N.T., Chan S.H. (2011). A review on membraneless laminar flow-based fuel cells. Int. J. Hydrogen Energy.

[3] Frank M., Erdler G., Frerichs H.P., Muller C., Reinecke H. (2008). Chip integrated fuel cell accumulator. J. Power Sources.

[4] Moore C.W., Li J., Kohl P.A. (2005). Microfabricated fuel cells with thin-film silicon dioxide proton exchange membranes. J. Electrochem. Soc..

[5] Moghaddam S., Pengwang E., Jiang Y.B., Garcia A.R., Burnett D.J., Brinker C.J. (2010). An inorganic–organic proton exchange membrane for fuel cells with a controlled nanoscale pore structure. Nat. Nanotechnol..

[6] Pattekar A.V., Kothare M.V. (2004). A microreactor for hydrogen production in micro fuel cell applications. J. Microelectromech. Syst..

[7] Moghaddam S., Pengwang E., Masel I.R., Shannon A.M. (2008). A self-regulating hydrogen generator for micro fuel cells. J. Power Sources.

[8] Yeom J., Jayashree R.S., Rastogi C., Shannon M.A., Kenis P.J.A. (2006). Passive direct formic acid microfabricated fuel cells. J. Power Sources.

[9] Paust N., Krumbholz S., Munt S., Müller C., Koltay P., Zengerle R. (2009). Self-regulating passive fuel supply for small direct methanol fuel cells operating in all orientations. J. Power Sources.

[10] Desheng D.M., Kim C.J. (2009). An active micro-direct methanol fuel cell with self-circulation of fuel and built-in removal of CO2 bubbles. J. Power Sources.

[11] Ji F., Yang L., Sun H., Wang S., Li H., Jiang L. (2017). A novel method for analysis and prediction of methanol mass transfer in direct methanol fuel cell. Energy Convers. Manag..

[12] Hollinger A.S., Doleiden D.G., Willis M.G., DeLaney S.C., MBurbules M.B., Miller K.L., Argun N. (2018). Model-based analysis of thermal and geometrical effects in a microscale methanol fuel cell. Int. J. Hydrogen Energy.

[13] Nguyen N.T., Chan S.H. (2006). Micromachined polymer electrolyte membrane and direct methanol fuel cells—A review. J. Micromech. Microeng..

[14] Tanveer M., Kim K.Y. (2018). Performance analysis of microfluidic fuel cells with various inlet locations and multiple compartments. Energy Convers. Manag..

[15] Montesinos P.O.L., Yossakda N., Schmidt A., Brushett F.R., Pelton W.E., Kenis P.J.A. (2011). Design, fabrication, and characterization of a planar, silicon-based, monolithically integrated micro laminar flow fuel cell with a bridge-shaped microchannel cross-section. J. Power Sources.

[16] Tanveer M., Kim K.Y. (2017). Effects of geometric configuration of the channel and electrodes on the performance of a membraneless micro-fuel cell. Energy Convers. Manag..

[17] Shen L.L., Zhang G.R., Venter T., Diesalski M., Bastian J.M.E. (2019). Towards best practices for improving paper-based microfluidic fuel cells. Electrochim. Acta.

[18] Ferrigno R., Stroock A.D., Clark T.D., Mayer M., Whitesides G.M. (2002). Membraneless vanadium redox fuel cell using laminar flow. J. Am. Chem. Soc..

[19] Kjeang E., Djilali N., Sinton D. (2009). Microfluidic fuel cells: A review. J. Power Sources.

[20] Tominaka S., Ohta S., Obata H., Momma T., Osaka T. (2008). On-chip fuel cell: Micro direct methanol fuel cell of an air-breathing, membraneless, and monolithic design. J. Am. Chem. Soc..

[21] Kenis P.J.A., Ismagilov R.F., Whitesides G.M. (1999). Microfabrication inside capillaries using multiphase laminar flow patterning. Science.

[22] Choban E.R., Markoski L.J., Wieckowski A., Kenis P.J.A. (2004). Microfluidic fuel cell based on laminar flow. J. Power Sources.

[23] Choban E.R., Markoski L.J., Stoltzfus J., Moore J.S., Kenis P.J.A. (2002). Microfluidic Fuel Cells that Lack a Polymer Electrolyte Membrane. Power Sources Proc..

[24] Sprague I.B., Byun D., Dutta P. (2010). Effects of reactant crossover and electrode dimensions on the performance of a microfluidic based laminar flow fuel cell. Electrochim. Acta.

[25] Chang M.H., Chen F., Fang N.S. (2006). Analysis of membraneless fuel cell using laminar flow in a Y-shaped microchannel. J. Power Sources.

[26] Park H.B., Lee K.H., Sung H.J. (2013). Performance of H-shaped membraneless micro fuel cells. J. Power Sources.

[27] Bazylak A., Sinton D., Djilali N. (2005). Improved fuel utilization in microfluidic fuel cells: A computational study. J. Power Sources.

[28] Khabbazi A.E., Richards A., Hoorfar M. Numerical analysis of the effect of different channel geometries and electrode materials on the performance of microfluidic fuel cells. Proceedings of the ASME 2010 8th International Conference on Fuel Cell Science, Engineering and Technology.

[29] Khabbazi A.E., Richards A.J., Hoorfar M. (2010). Numerical study of the effect of the channel and electrode geometry on the performance of microfluidic fuel cells. J. Power Sources.

[30] Li L., Fan W., Xuan J., Leung M.K.H., Zheng K., She Y. (2017). Optimal design of current collectors for microfluidic fuel cell with flow-through porous electrodes: Model and experiment. Appl. Energy.

[31] Hanapi I.H., Kamaruddin S.K., Zainoodin A.M., Hasran U.A. (2019). Membrane-less micro fuel cell system design and performance: An overveiw. Int. J. Energy Res..

[32] Shaegh S.A.M., Nguyen N.T., Chan S.H. (2010). An air-breathing microfluidic formic acid fuel cell with a porous planar anode: Experimental and numerical investigations. J. Micromech. Microeng..

[33] Krishnamurthy D., Johansson E.O., Lee J.W., Kjeang E. (2011). Computational modeling of microfluidic fuel cells with flow-through porous electrodes. J. Power Sources.

[34] Montesinos P.O.L., Desai A.V., Kenis P.J.A. (2014). A three-dimensional numerical model of a micro laminar flow fuel cell with a bridge-shaped microchannel cross-section. J. Power Sources.

[35] Tanveer M., Kim K.Y. (2018). Performance analysis of a micro laminar flow fuel cell with multiple inlets of a bridge-shaped microchannel. J. Power Sources.

[36] Ni M., Leung M.K.H., Leung D.Y.C. (2007). Parametric study of solid oxide fuel cell performance. Energy Convers. Manage..

[37] COMSOL AB (2012). COMSOL Multiphysics Reference Guide.

[38] Archie G.E. (1947). Electrical resistivity an aid in core-analysis interpretation. AAPG Bull..

[39] Archie G.E. (1950). Introduction to Petrophysics of Reservoir Rocks. AAPG Bull..

